# The development of a randomised controlled trial testing the effects of an online intervention among school students at risk of suicide

**DOI:** 10.1186/1471-244X-14-155

**Published:** 2014-05-27

**Authors:** Jo Robinson, Sarah Hetrick, Georgina Cox, Sarah Bendall, Alison Yung, Hok Pan Yuen, Kate Templer, Jane Pirkis

**Affiliations:** 1 Orygen Youth Health Research Centre, Centre for Youth Mental Health, University of Melbourne, Locked Bag 10, Parkville, VIC 3052, Australia; 2 Institute of Brain, Behaviour and Mental Health, University of Manchester, Oxford Road, Manchester M13 9PL, UK; 3 Melbourne School of Population and Global Health, University of Melbourne, Level 4, 207 Bouverie Street, Parkville, VIC 3010, Australia

**Keywords:** Suicide, Cognitive behavioural therapy, Adolescents, Schools, Internet, Randomised controlled trial, Youth, Online

## Abstract

**Background:**

Suicide-related behaviour among young people is of significant concern, yet little is known regarding the effectiveness of interventions designed to reduce risk among this population. Of those interventions that have been tested, cognitive-behavioural therapy appears to show some promise among young people with suicidal ideation. Internet-based interventions are becoming increasingly popular and have shown some effect in preventing and treating depression and anxiety in young people. However, to date there are no randomised controlled trials examining the impact of Internet-based Cognitive Behavioural Therapy among suicidal youth.

**Methods/design:**

This is a randomised controlled trial testing the effects of Internet-based cognitive-behavioural therapy among suicidal high school students who have sought help from the school wellbeing team. The intervention comprises 8 modules of Cognitive Behavioural Therapy delivered online. The study has a staggered, two-year recruitment phase and participants are assessed at baseline, post intervention and 12 weeks later.

**Discussion:**

If effective the program has the ability to be readily adapted and delivered to a range of populations in a range of settings, at relatively little cost. It can also be adapted for mobile applications.

**Trial registration:**

ACTRN12613000864729. Date registered: 05/08/2013

## Background

Suicide-related behaviours (SRB), including suicide attempts (SA) and suicidal ideation are common among young people. Up to 24% of 12–17 year-olds have reported suicidal ideation, and 7-11% have reported a 12-month prevalence of suicide attempt [1]. These behaviours are one of the greatest concerns for Australian young people [2] and are associated with a range of negative outcomes including completed suicide and premature mortality via other causes [3,4]. The prevention of suicide, and the development of a strategic research agenda targeting interventions for suicidal youth have both been cited as national priorities [5,6], yet there remains a lack of high quality intervention research for suicidal individuals [7], including youth [8].

Depression is the most common risk factor for SRB. Suicidal youth are six times more likely to have a psychiatric disorder than non-suicidal youth [9-11]. The most common disorder is depression, with between 60 and 80% of young people having a diagnosis of depression at the time of a SA [12]. Hopelessness has long been linked to increased suicide risk, including among young people, and is believed to mediate the relationship between depression and suicide-related behaviour [13,14].

Notwithstanding this, not all suicidal young people experience symptoms of depression; therefore interventions that specifically target suicidal young people are required. Good evidence exists regarding the treatment of youth depression [15], but there is limited knowledge regarding effective interventions for suicide-related behaviour [7,8]. Of the psychological approaches that have been tested Cognitive Behavioural Therapy (CBT) appears to be the most promising in terms of its ability to reduce suicidal ideation among adolescents and young adults, however further research is required [8].

CBT is used extensively in the treatment of adolescent depression [16] and is recommended as a first-line treatment for depressed youth [17]. Components frequently used with depressed adolescents include: basic psycho-education; pleasant activity scheduling; cognitive restructuring; problem-solving; and relaxation training [16].

In response to the growing popularity of electronic means of communication, in particular among youth, CBT interventions are now routinely delivered via the Internet. Internet-based CBT has been shown to be an effective and cost-effective form of treatment for depression and anxiety among adults [18-24], and has the potential to be more accessible and less stigmatising than traditional, face-to-face models of therapy [25,26]. It has also been shown to have the potential both to prevent and reduce symptoms of depression and anxiety in adolescents [27,28].

Adherence has been highlighted as a particular issue with online interventions [29]. There is evidence to suggest that programs that are password protected, practitioner prescribed and supported, tend to have higher rates of adherence, lower rates of attrition and better treatment outcomes than open access sites [23,30].

Given that practitioner involvement appears to improve outcomes, and that school wellbeing staff are considered helpful by students when it comes to mental health-related difficulties [31], the development of an Internet-based CBT program that can be delivered by school wellbeing staff is a logical next step. Indeed schools are an obvious and accepted environment for implementing suicide prevention initiatives [11,32-34].

Yet, despite the potential benefits of Internet-based CBT, there is virtually no research into the impact of Internet-based CBT on SRB. To date only one study specifically set out to test the effects of an Internet-based program among suicidal adults, and reported a reduction in SI [35], and two studies testing online interventions for depression also demonstrated a reduction in suicide-related outcomes [36,37]. No studies have targeted suicidal youth.

In response to this, we have developed and piloted an Internet-based program for school students at risk of suicide called Reframe-IT. Reframe-IT has been specifically designed for young people, and unlike other Internet-based programs, is designed to be delivered and supported, by school wellbeing staff.

The program has been piloted with 21 young people from nine schools. Findings show a reduction in suicidal ideation, depressive symptoms and hopelessness [38], and an increase in problem solving and coping skills (Hetrick et al, forthcoming) over the course of the program. The data also show that the modules do not induce either distress or suicidal ideation, and overall participants report finding the program enjoyable and say that they would recommend it to a friend [39]. The pilot study was small and uncontrolled, therefore the program requires testing in a randomised controlled trial to confirm its effectiveness.

## Methods

### Aims

The primary aim of this study is to examine whether or not participation in the Reframe-IT program leads to reduced: 1) suicidal ideation. Additional aims are to examine its impact on: 2) symptoms of depression, and 3) levels of hopelessness, among participating students, and to determine if it leads to: 4) increased confidence 5) increases in perceived skill, and 6) changes in practice among school wellbeing staff (in particular with regard to their use of Internet-based programs and resources with at-risk students).

### Study design

The student-related aims listed above will be addressed via a randomised-controlled trial. The study has a staggered two-year recruitment phase and involves the delivery of eight modules of CBT delivered over a 10-week intervention period. Students are followed up post intervention and again 12 weeks later. The study has been designed in order that the CONSORT guidelines [40] can be met when reporting the trial.

The aims related to school staff will be assessed using a pre-test/post-test design.

The study team comprises a project coordinator (JR), a research therapist (SH); two research assistants; a clinical psychologist (SB), and a statistician (HPY).

### Recruitment

All secondary schools in the study catchment area (north west metropolitan Melbourne) will be invited to participate. Up to 28 of the responding schools will be selected on a first-come first-served basis. Schools will be recruited in a staggered manner. Participants (n = 170) will also be recruited in a staggered manner, over a two-year period. In order to meet the inclusion criteria students must be aged 14 to 18 and report any level of SI in the past month. They must also provide written consent from themselves and their parents/guardians. Exclusion criteria are an intellectual disability, psychotic symptoms and/or inability to speak English. Participants will be recruited by members of the student wellbeing team at each school. All students who present to the school wellbeing staff member and meet the inclusion criteria will be asked if they are interested in hearing more about the study by that staff member. They will also be given a brief information sheet explaining the study, which they can read and share with their parents/guardian. If the student indicates that they would like to take part they are given a detailed information and consent form to take home to be signed.

**Figure 1:**
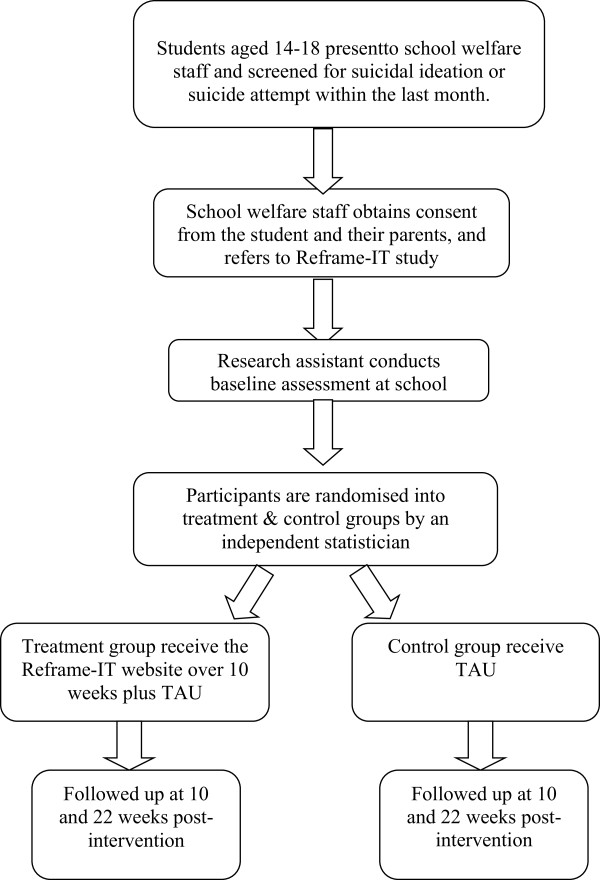
Study flow-chart.

Withdrawal from the study will occur if participation in the study interferes with clinical management of risk, or if the participant develops psychotic symptoms.The participant flow chart is shown in Figure 1.

### Intervention

The Reframe-IT intervention comprises eight modules to be delivered during the 10-week intervention period. Each participant will have access to his or her own personalised webpage accessed via secure login. For safety reasons, the program will be administered in the young person’s school by the student wellbeing staff member. Once each individual module has been completed in the presence of the school staff member, participants will be able to access it from home, 24 hours a day. The program has no social networking function.

The program takes the form of an adult ‘host’ character that delivers the therapy verbally, and a series of video diaries made by young people. There are two activities per week plus homework. The site has a message board through which the participant can communicate with the research-therapist; a series of factsheets covering a range of related topics, including managing suicidal thoughts; plus downloadable relaxation MP3s. As the weeks progress additional items are added to the site, (e.g. an activity diary). Finally there is a ‘Getting help’ tab, which lists a range of local and national helplines and services that the participant can access if in crisis.

The eight modules incorporate standard CBT approaches commonly used with young people but have a specific focus on suicidal thinking and behaviours [16]: engagement and agenda setting; emotional recognition and distress tolerance; identification of negative automatic thinking; behavioural activation - help-seeking and activity scheduling (including relaxation techniques); problem solving; detecting and challenging problematic thinking, and cognitive restructuring.

Practitioner involvement will be two-fold. First, the SWC will facilitate delivery of the program (i.e. setting up appointments; managing Internet issues; and remaining in the vicinity while the participant views the program). They will also check responses to a weekly suicide screen and respond accordingly. Second, there will be involvement from the research therapist (SH) who will remain un-blinded. She will check completed activities and respond with personalised but standardised messages. She will also check the message board daily and respond accordingly.

### Control intervention

The control group will receive Treatment As Usual only. This will be monitored via questionnaire, and a weekly client contact sheet completed with each school.

### Outcome measurements

#### Student outcomes

Participants will be assessed prior to delivery of the intervention (baseline), immediately post-intervention (10 weeks) and 12 weeks later. The research assistant will administer each of the following measures at each time point, with the exception of the evaluation questionnaire, which is only administered post-intervention. All assessments will be conducted at school.

The primary outcome is reduced suicidal ideation at post-intervention and follow-up, measured by the Suicidal Ideation Questionnaire (SIQ), a 30-item self-report measure designed to assess suicidal ideation in adolescents. It has been validated with clinical and non-clinical populations and shown to have high levels of internal consistency and test-retest reliability, and high levels of construct and criterion validity [41,42].

Other outcomes to be measured include:

• Suicidal acts - using a series of specifically-designed questions.

• Depressive symptoms - using the clinician-rated Children's Depression Rating Scale-Revised (CDRS-R) [43], and the Reynolds Adolescent Depression Scale-2 (RADS-2) [44]. The CDRS-R is a semi-structured interview schedule originally designed for use with children but also successfully used with adolescents. It rates depressive symptoms across 17 domains including difficulties with schoolwork, social withdrawal, appetite and sleep disturbance, fatigue, guilt and suicidal ideation. The first 14 items are rated on the basis of responses to interview questions from the young person, and are rated for the past two weeks and currently. The remaining three symptom areas (depressed facial affect, listless speech and hypoactivity) are rated by the clinician on the basis of the participant's non-verbal behaviour. Each symptom is graded on a 5 or 7-point scale with increasing scores indicating increasing severity of symptoms. The total score, or CDRS-R score, is the sum of all 17-item scores and the instrument has a range of 17-113. This scale is used widely, has adequate internal reliability, good test-retest reliability, good to excellent inter-rater reliability and is sensitive to treatment effects [43,45]. The RADS-2 is 10-item brief screening measure for the assessment of current depressive symptoms in adolescents aged between 11 and 20. It can be used in clinical or non-clinical populations and takes around 2-3 minutes to complete. Items assess mood or reduced affect, loneliness, social withdrawal, sadness, self-harm, self-reproach, self-worth, anger/irritability, loss of interest and helplessness. Participants are asked to rate how often they experience each item on a 4-point Likert scale ranging from ‘almost never’ to ‘most of the time’. Responses are rated from 1-4, giving a possible total score range of 10-40 points, with higher scores indicating greater symptom severity. The instrument includes one reverse-scored item that is worded in a positive manner, so that reversing the individual’s score represents greater depression. A cut-off score of 26 on this measure indicates clinically significant symptomatology. The RADS-2 has demonstrated strong internal consistency and a moderately high level of test-retest reliability among school students. It has also demonstrated strong evidence of criterion validity when assessed against both clinical interview and self report measures of depression, and strong construct and clinical validity [44,46].

• Hopelessness will be measured using the Beck Hopelessness Scale [47]. This is a self-report scale consisting of 20 true or false items, 9 or which are keyed ‘false’ and 11 are keyed ‘true’. For every statement, each response is assigned a score of 0 or 1, and the total ‘hopelessness score’ was the sum of the scores on the individual items. Thus, the possible range of scores was from 0 to 20, with a higher score indicating a greater degree of hopelessness. A cut-off score of 9 on the scale has been found to be predictive of eventual suicide among clinical samples [48]. This scale has been found to have a high degree of internal consistency and good test-retest reliability and good construct and concurrent validity.

• Anxiety will be measured using the Multidimensional Anxiety Scale for Children [49]. This is a 39-item self-report instrument that assesses the major aspects of anxiety in young people. Items are distributed across four dimensions: physical symptoms, harm avoidance, social anxiety and panic. Participants are asked think about how they have been feeling recently and to rate each item on a 4-point scale of 0-3 ranging from this is ‘never true about me’ to this is ‘often true about me’. Scores are derived from totals on each subscale together with those from a total anxiety scale, an anxiety disorders index and an inconsistency scale. Detailed instructions on scoring and interpretation are provided in the user manual. The MASC has shown adequate internal consistency and satisfactory test-retest reliability. It also has strong discriminant validity and moderate construct validity [49].

• Problem-solving skills will be assessed using the Negative Problem-Oriented Questionnaire [50]. This is a 12-item self-report measure that assesses negative problem orientation. Participants are asked to rate their responses on a 5-point scale with responses ranging from 1 – ‘not at all true of me’ to 5 – ‘extremely true of me’. The measure is unifactorial with no inverted questions. A higher score indicates a greater degree of negative beliefs concerning one’s problems and problem-solving ability. The scale has demonstrated excellent internal consistency, good test–retest reliability at five weeks, and both convergent and discriminant validity when measured against self-reported pessimism, depression, anxiety, and problem-solving ability.

• The Cognitive-behavioural Therapy Skills Questionnaire [51] will be used to test which components of the CBT appear to be most active. This 16-item scale measures the use of cognitive restructuring and behavioural activation skills i.e. changes in avoidance/behavioural control and changes in cognitive style. Respondents rank each item on a 5-point Likert scale from 1 (I don’t do this) to 5 (I always do this). It is a relatively new measure but it has been shown to be both reliable and valid.

• Distress will be measured using the abbreviated version of the Profile of Moods States (POMS) [52,53]. The POMS is a 14-item self-administered checklist measuring transient mood states. This measure has been used previously with adolescents for this purpose [54] and has been demonstrated to be sensitive to short-term change [52,53]. The POMS data can be used to produce the following six subscales: anger, depression, fatigue, tension, confusion and vigour. The score of each item ranges from 0-4. Each subscale is scored by adding up the scores of the related items and a higher score reflects greater presence of that item.

• At baseline, demographic information will be collected using a specifically designed questionnaire, and the first four subscales of the Comprehensive Assessment of At-risk Mental State (CAARMS) [55], to screen for psychosis. The CAARMS is a semi-structured clinician administered interview schedule that was specifically designed to assess for signs of psychosis in young people. It includes eight subscales that measure disorders of thought content; perceptual abnormalities; conceptual disorganisation; motor changes; concentration and attention; emotion and affect; subjectively impaired energy and impaired tolerance to normal stress. Scores for each subscale range from 0-6 with a higher score indicating greater likelihood of a psychotic disorder being present. It has been found to have good to excellent concurrent, discriminant and predictive validity and excellent inter-rater reliability.

To test for possible confounders the following will also be assessed:

• Treatment history - using a specifically developed questionnaire.

• Substance use, via the Substances and Choices Scale [56], which is a one-page self-report questionnaire designed for young people aged 13-18 years and takes about 5 minutes to complete. It has three sections. The first section records the number of occasions the young person has used a variety of substances in the last month. The second section measures both substance use related symptoms and substance related harm. Scoring this section yields the ‘SACS difficulties score’ from 0 to 20. This score can be used to screen or measure change through a treatment episode. The third section asks about tobacco use. It has been shown to demonstrate good reliability and validity.

• Help seeking via the General Help Seeking Scale [57]. The General Help Seeking Scale measures help-seeking intentions, appraising both formal and informal sources. Participants rate help-seeking intentions ranging from 1 (‘extremely unlikely’) to 7 (‘extremely likely’) for each help source option including ‘no one’. Higher scores indicate higher intentions.

Participant satisfaction and the extent to which they find the program acceptable will be measured using a specifically designed measure at post-intervention.

All outcome measures with the exception of the CDRS-R will be administered online. Research staff will receive training in the administration of the CDRS-R by one of the two clinical psychologists on the study team. All research staff will also be trained in the assessment of suicide risk.

#### School staff outcomes

These will be measured using a specifically designed questionnaire, based on those previously used by this research team [58]. This will be administered prior to the beginning of each school’s involvement in the study and at the end of the intervention period. If a staff member leaves the school before the end of the study they will be asked to complete the questionnaire before their departure. The questionnaire will assess attitudes, confidence; perceived skill and changes in practice.

All outcome data will de-identified and stored on a password-protected database located on a secure server housed by Orygen Youth Health Research Centre.

### Randomisation/treatment allocation

Participants (n = 170) will be recruited via each school’s student wellbeing coordinator. Once written consent is obtained from a potential participant, a baseline assessment will be conducted to ascertain his or her eligibility. The study coordinator (JR) will randomise eligible participants into either the treatment or control group using a randomisation list prepared by an independent statistician. The randomisation list (generated using random number generator computer software) is stratified by school and is incorporated into an online randomisation computer program. Immediately after each randomisation, the relevant school wellbeing staff member will be automatically notified of treatment allocation via email. They will then administer the appropriate treatment to the participant.

### Blinding and treatment fidelity

Because of the nature of the intervention (i.e. it is to be delivered by school staff) it is not possible for school staff to be blind to intervention. In addition, the study coordinator (JR) and the two study psychologists (SH and SB) will remain unblinded in order that randomisation can be conducted and to enable the website to be moderated. However the research assistants conducting outcome assessments and the statistician conducting the analysis will remain blinded to treatment allocation until study completion.

With regard to treatment fidelity, a study manual has been developed to facilitate this across sites. However it is acknowledged that the same school staff will be administering both the treatment and control interventions to students. Therefore there is the possibility of some contamination, in terms of school wellbeing staff incorporating some of the techniques from the CBT program into their work with students allocated to the control condition. However we consider this to be unlikely. Although school staff will be responsible for administering the program, they will not be required to supervise the student closely whilst they watch the program. Rather their role will be to set up appointments with the students, to remain in the vicinity whilst each module is completed, and to check the suicidal ideation screen at the end of each module. However in order to assess this we will ask all school wellbeing staff to complete a short checklist at the end of each session with a Reframe-IT student that will record the length and content of each session.

### Statistical analysis

#### Student outcomes

At each follow-up time point the general linear model will test for the treatment factor, i.e. compare the intervention and control groups for each outcome measure. Corresponding baseline values of each outcome measure will be used as the covariate. In addition, possible effect of site will be examined by introducing a school factor in the analysis. Multi-level modeling will be used to compare the two groups in terms of the trend over time for each outcome. Both the last observation carried forward and multiple imputation techniques will be considered if missing data are substantial. Interim analysis will be conducted twice yearly, or upon request to facilitate the safety monitoring of the project.

Adherence will be measured using data automatically collected by the system (e.g. number of times each participant logs onto the site, amount of time spent on the site, proportion of activities completed).

#### School staff outcomes

Descriptive statistics (mean, SD, median) will be used to gauge the level of confidence and perceived ability at baseline and post-intervention. Paired *t*-test will be used to test if there is a significant change between baseline and follow-up. The general linear model will also be used to examine if the changes are related to possible covariates such as baseline levels and previous training.

### Effect size and statistical power

The primary outcome of interest is changes in suicidal ideation from baseline measured at 10 and 22 weeks. This will be measured by the SIQ. No previous studies have tested the effects of an Internet-based CBT intervention on suicidal ideation, however one previous study [59] has reported moderate to large effect sizes in terms of reduced suicidal ideation, for face-to-face CBT among young people. If we assume that alpha is set at 0.05 and power at 0.80, then a sample size of 58 is required for each of the two groups (total n = 116) to detect a medium effect size. This is based on using the general linear model to compare the intervention and control groups with the baseline values of an outcome measure as the covariate. It is assumed (conservatively) that the covariate would explain 10% of the variance in the dependent variable. Using data from the pilot study, the intra-cluster correlation corresponding to the schools is estimated to be 0.023, which in turn gives an estimate of 1.1 for the design effect. So in order to take account of the clustering effect of the schools, the sample size needs to be inflated to 128 (116 × 1.1).

A previous RCT conducted by the study team with a similar population had a dropout rate of 24% [60]. The proposed study differs in that the intervention will be delivered by school staff and all follow-up assessments will be conducted at school. It will also employ a shorter follow-up period, however we have estimated a dropout rate of 24% meaning that we need to recruit 169 students (128/0.76) into the study. We estimate that we can recruit about 6 students from each school, so we need to recruit 28 schools. Based on data obtained from a pilot study conducted prior to developing the RCT [39] we consider these figures to be feasible.

Every effort will be made to promote participant retention, including close liaison with school staff, maximum flexibility on the part of research staff conducting follow-up assessments, and by employing a number of methods to contact students for follow-up assessments. These will include contacting the participant via the school wellbeing coordinator in the first instance, but also by contacting them directly via phone or email and contacting them via their parents/guardians if necessary.

### Ethics and safety

Ethical approval has been obtained from the University of Melbourne Human Research and Ethics Committee.

#### Protocols and supervision

Clear and detailed safety protocols have been developed for this study detailing how undue risk will be determined and how the research team will respond. The research assistants will receive training in the administration of the measures and in the assessment of suicide risk. The research assistant will make contact with the school wellbeing staff member once each assessment is completed. If the research assistant and the school are concerned about the participant the project coordinator will be informed and, where necessary, an immediate referral will be made to an appropriate service. Fortnightly supervision meetings with the research therapist and the clinical psychologist will be held, during which all cases will be presented and any diagnostic and/or risk issues discussed.

#### Weekly screening

In addition, participants will complete a weekly suicidal ideation screen, which will be checked by the school wellbeing staff member at the end of each session. If a participant indicates current suicidal ideation, a risk assessment will be conducted. If they are determined to be at elevated risk then the staff member will be required to follow the school’s safety protocols. They will also receive an automated email prompting them to follow these procedures.

#### Website moderation

The website will be moderated on a daily basis five days a week during the school term by the clinical psychologist and clear procedures have been established to manage any indication of risk. It will be made clear to all participants that the website will not be moderated seven days a week, 24 hours per day, therefore if urgent messages are posted on the site they may not be seen for a couple of days. The website will include information about appropriate sources of help in a crisis and participants will be encouraged to use these contacts when necessary instead of contacting the research team.

An independent safety advisory committee will oversee all safety procedures. If at any point during the trial the committee is concerned about the welfare of participants as a result of their participation in the study, they will have the capacity to suspend the trial.

All data collected will be stored securely and according to the University of Melbourne’s Policy on the Management of Research Data and Records.

## Discussion

This paper describes the development of a randomised controlled trial that aims to examine the efficacy of an online CBT intervention to reduce suicide risk in secondary school students.

As noted above, there is limited evidence regarding the effectiveness of interventions for this population. Evidence from randomised controlled trials is particularly lacking [8], and although there are a range of valid reasons for the limited number of controlled trials in the field of suicidology [61], they remain the gold standard means of testing the effectiveness of interventions. Therefore RCTs are required if we are to establish effective interventions for at-risk youth.

One of the reasons postulated for the lack of randomised controlled trials with this population relate to sample size issues [61]. Others relate to ethical concerns surrounding withholding potentially efficacious interventions to youth at risk, as well as fears that asking young people about suicide-related thoughts or behaviours will cause distress and increase subsequent risk. Together these factors have meant that suicidal youth are often excluded from research into face-to-face therapy [62], as well as from studies testing Internet-based interventions [22].

However, research has indicated that conducting research with this population can be both safe and acceptable [31,39,54,63]. In addition, Internet-based interventions such as this have the capacity to reach large numbers of people [64]. Yet, despite this, and despite the extent of the problem of suicide-related behaviours in young people, to our knowledge, this project is the first internationally to test an Internet-based intervention specifically among young people at risk of suicide.

This program differs from other Internet-based CBT programs in that it uses a series of video diaries as opposed to being text-based. It also specifically addresses SRB within the modules, and it is practitioner-administered. It was noted above that programs that are practitioner administered tend to have higher rates of adherence and engagement than open access programs [23,30]. However this does mean that the program relies on young people seeking help from a school wellbeing staff member and it makes the program more resource intensive than other programs. However, given the fact that participants in this program are at risk of suicide the benefits associated with practitioner involvement are believed to outweigh the potential drawbacks.

We do acknowledge that attrition may still be a problem. Every effort will be made to promote participant retention, including close liaison with school staff, maximum flexibility on the part of research staff conducting follow-up assessments, and by employing a number of methods to contact students for follow-up assessments. These will include contacting the participant via the school wellbeing coordinator in the first instance, but also by contacting them directly via phone or email and contacting them via their parents/guardians if necessary.

The program is highly transferable and easy to implement, meaning that, if effective it could readily be adapted and made available to a range of high-risk populations across a number of settings (either as an add-on or alternative to face-to-face treatment). It also has the capacity to reach rural and remote areas, where access to services can be limited and to be adapted for mobile applications. Indeed, as much of the cost of such an intervention is associated with the development and initial testing, once evaluated it has the potential to be adapted and rolled out to large numbers of people with ease and at relatively little cost.

## Competing interests

The authors declare that they have no competing interests.

## Authors’ contributions

Authors JR, SH and JP were all involved in conception of the study, intervention development and made substantial contributions to the study design. HPY contributed to study design and has responsibility for al statistical analysis. In addition SH, SB have made significant contributions training and clinical supervision. Authors AY, GC and KT have been involved in piloting the study and developing all study protocols. All authors have been involved in drafting the manuscript and have seen and approved the final version.

## Pre-publication history

The pre-publication history for this paper can be accessed here:

http://www.biomedcentral.com/1471-244X/14/155/prepub

## References

[B1] Nock MK Borges G Bromet EJ Cha CB Kessler RC Lee S Suicide and suicidal behavior Epidemiol Rev 2008 30 1 133 154 10.1093/epirev/mxn002 18653727 PMC2576496

[B2] Mission-Australia National Survey of Young Australians, Key and Emerging Issues 2009 Sydney, New South Wales: Mission-Australia

[B3] Hawton K Fagg J Suicide, and other causes of death, following attempted suicide Br J Psychiatry 1988 152 359 366 10.1192/bjp.152.3.359 3167371

[B4] Suominen K Isometsa E Suokas J Haukka J Achte K Lonnqvist J Completed suicide after a suicide attempt: a 37-year follow-up study Am J Psychiatry 2004 161 3 562 563 10.1176/appi.ajp.161.3.562 14992984

[B5] Commonwealth-Government The hidden toll: suicide in Australia. Report of the Senate Community affairs Reference Committee 2010 Canberra: Commonwealth of Australia

[B6] Commonwealth-Government Before it’s too late: report on early intervention programs aimed at preventing youth suicide 2011 Canberra: Commonwealth of Australia

[B7] Robinson J Pirkis J Krysinska K Niner S Jorm A Dudley M Schindeler E De Leo D Harrigan S Research priorities in suicide prevention in Australia. A comparison of current research efforts and stakeholder-identified priorities Crisis 2008 29 4 180 190 19069610 10.1027/0227-5910.29.4.180

[B8] Robinson J Hetrick S Martin C Preventing suicide in young people: systematic review ANZJP 2011 45 1 3 26 21174502 10.3109/00048674.2010.511147

[B9] Patton G Harris R Carlin J Hibbert M Coffey C Schwartz M Bowes G Adolescent suicide behaviours: a population-based study of risk Psychol Med 1997 27 715 724 10.1017/S003329179600462X 9153691

[B10] Foley DL Goldston DB Costello EJ Angold A Proximal psychiatric risk factors for suicidality in youth: the Great Smoky Mountains Study Arch Gen Psychiatry 2006 63 9 1017 1024 10.1001/archpsyc.63.9.1017 16953004

[B11] Hawton K Rodham K Evans E Weatherall R Deliberate self-harm in adolescents: self-report survey in schools in England BMJ 2002 325 1207 1211 10.1136/bmj.325.7374.1207 12446536 PMC135492

[B12] Cash SJ Bridge JA Epidemiology of youth suicide and suicidal behavior Curr Opin Pediatr 2009 21 5 613 619 10.1097/MOP.0b013e32833063e1 19644372 PMC2885157

[B13] Kwok S Shek D Hopelessness, parent-adolescent communication, and suicidal ideation among chinese adolescents in Hong Kong Suicide Life-Threat 2010 40 3 224 233 10.1521/suli.2010.40.3.224 20560744

[B14] Kazdin A French N Unis A Esveldtdawson K Sherick R Hopelessness, depression, and suicidal intent among psychiatrically disturbed inpatient children J Consult Clin Psych 1983 51 4 504 510 10.1037//0022-006x.51.4.504 6619356

[B15] Compton SN March JS Brent D Albano AM Weersing R Curry J Cognitive-behavioral psychotherapy for anxiety and depressive disorders in children and adolescents: an evidence-based medicine review J Am Acad Child Adolesc Psychiatry 2004 43 8 930 959 10.1097/01.chi.0000127589.57468.bf 15266189

[B16] Weersing VR Rozenman M Gonzalez A Core components of therapy in youth Behav Modif 2009 33 1 24 47 18955540 10.1177/0145445508322629

[B17] McDermott B Baigent M Chanen A Fraser L Graetz B Hayman N Newman L Parikh N Peirce B Proimos J Smalley T Spence SH Clinical practice guidelines: depression in adolescents and young adults 2010 Melbourne: Beyondblue: the national depression initiative

[B18] Gerhards SAH de Graaf LE Jacobs LE Severens JL Huibers MJH Arntz A Riper H Widdershoven G Metsemakers JFM Evers SMAA Economic evaluation of online computerised cognitive-behavioural therapy without support for depression in primary care: randomised trial Br J Psychiatry 2010 196 4 310 318 10.1192/bjp.bp.109.065748 20357309

[B19] Griffiths KM Farrer L Christensen H The efficacy of internet interventions for depression and anxiety disorders: a review of randomised controlled trials Med J Aust 2010 192 11 S4 S11 20528707 10.5694/j.1326-5377.2010.tb03685.x

[B20] Kaltenthaler E Parry G Beverley C Computerized cognitive behavioural therapy: a systematic review Behav Cogn Psychother 2004 32 01 31 55 10.1017/S135246580400102X

[B21] Proudfoot J Ryden C Everitt B Shapiro DA Goldberg D Mann A Tylee A Marks I Gray JA Clinical efficacy of computerised cognitive-behavioural therapy for anxiety and depression in primary care: randomised controlled trial Br J Psychiatry 2004 185 1 46 54 10.1192/bjp.185.1.46 15231555

[B22] Titov N Status of computerized cognitive behavioural therapy for adults ANZJP 2007 41 2 95 114 17464688 10.1080/00048670601109873

[B23] Spek V Cuijpers P Nyklícek I Riper H Keyzer J Pop V Internet-based cognitive behaviour therapy for symptoms of depression and anxiety: a meta-analysis Psychol Med 2007 37 03 319 328 10.1017/S0033291706008944 17112400

[B24] Kessler D Lewis G Kaur S Wiles N King M Weich S Sharp DJ Araya R Hollinghurst S Peters TJ Therapist-delivered internet psychotherapy for depression in primary care: a randomised controlled trial Lancet 2009 374 9690 628 634 10.1016/S0140-6736(09)61257-5 19700005

[B25] Cuijpers P van Straten A Andersson G Internet-administered cognitive behavior therapy for health problems: a systematic review J Behav Med 2008 31 2 169 177 10.1007/s10865-007-9144-1 18165893 PMC2346512

[B26] Berger M Wagner TH Baker LC Internet use and stigmatized illness Soc Sci Med 2005 61 8 1821 1827 10.1016/j.socscimed.2005.03.025 16029778

[B27] Richardson T Stallard P Velleman S Computerised cognitive behavioural therapy for the prevention and treatment of depression and anxiety in children and adolescents: a systematic review Clin Child Fam Psychol Rev 2010 13 3 275 290 10.1007/s10567-010-0069-9 20532980

[B28] Calear AL Christensen H Review of internet-based prevention and treatment programs for anxiety and depression in children and adolescents Med J Aust 2010 192 11 Suppl S12 S14 20528700 10.5694/j.1326-5377.2010.tb03686.x

[B29] Christensen H Griffiths K Farrer L Adherence in internet interventions for anxiety and depression: systematic review JMIR 2009 11 2 e13 19403466 10.2196/jmir.1194 PMC2762797

[B30] Cavanagh K Bennett-Levy J, Rishcards DA, Farrand P, Christensen H, Griffiths KM, Kavanagh DJ, Klein B, Lau MA, Proudfoot J, Ritterband L Turn on, tune in and (don't) drop out: engagement, adherence, attrition, and alliance with internet-based interventions Oxford Guide to Low Intensity CBT Interventions 2010 New York: Oxford University Press 227 232

[B31] Robinson J Gook S Pan Yuen H Hughes A Dodd S Bapat S Schwass W McGorry P Yung Y Depression education and identification in schools: an Australian-based study School Mental Health 2010 2 1 13 22 10.1007/s12310-009-9022-9

[B32] Lake AM Gould MS O'Connor RC, Platt S, Gordon J School-based strategies for youth suicide prevention International Handbook of Suicide Prevention: Research, Policy and Practice 2011 Chichester: John Wiley & Sons 507 530

[B33] Mental Health Foundation Truth hurts - report of the national inquiry into self-harm among young people 2006 London: Mental Health Foundation

[B34] Robinson J Pan Yuen H Martin C Hughes A Baksheev GN Dodd S Bapat S Schwass W McGorry P Yung AR Does screening high school students for psychological distress, deliberate self-harm or suicidal ideation cause distress and is it acceptable? An Australian based study Crisis 2011 32 5 254 263 21940259 10.1027/0227-5910/a000087

[B35] Van Spijker B Van Straten A Kerkhof A Effectiveness of online self-help for suicidal thoughts: Results of a randomised controlled trial PLoS One 2014 9 2 e90118 10.1371/journal.pone.0090118 24587233 PMC3937447

[B36] Christensen H Farrer L Batterham P Mackinnon A Griffiths KM Donker T The effect of a web-based depression intervention on suicide ideation: Secondary outcome from a randomised controlled trial in a helpline BMJ Open 2013 3 e002886 23811172 10.1136/bmjopen-2013-002886 PMC3696875

[B37] Watts S Newby JM Mewton L Andrews G A clinical audit of changes in suicide ideas with internet treatment for depression BMJ Open 2012 2 e001558 22983787 10.1136/bmjopen-2012-001558 PMC3467611

[B38] Robinson J Hetrick S Cox G Bendall S Yuen HP Yung A Pirkis J Can an internet-based intervention reduce suicidal ideation, depression and hopelessness among secondary school students: results from a pilot study Early Interv Psychiatry 2014 doi:10.1111/eip.12137 10.1111/eip.12137 24684946

[B39] Robinson J Hetrick S Cox G Bendall S Yung A Pirkis J The safety and acceptability of delivering an online intervention to secondary students at risk of suicide: findings from a pilot study Early Interv Psychiatry 2014 doi:10.1111/eip.12136 10.1111/eip.12136 24684927

[B40] Schulz KF Altman DG Moher D CONSORT Group CONSORT 2010 Statement: updated guidelines for reporting parallel group randomised trials BMJ 2010 340 c332 10.1136/bmj.c332 20332509 PMC2844940

[B41] Reynolds WM Suicidal Ideation Questionnaire: Professional manual 1988 Odessa, FL: Psychological Assessment Resources

[B42] Davis JM Suicidal Ideation Questionnaire J Psychoeduc Assess 1992 10 3 298 301 10.1177/073428299201000311

[B43] Poznanski EO Mokros HB Children’s Depression Rating Scale Revised (CDRS-R) 1996 Los Angeles: Western Psychological Services (WPS)

[B44] Reynolds WM Reynolds Adolescent Depression Scale Professional Manual 1987 Odessa, Florida: Psychological Assessment Resources

[B45] Myers K Winters NC Ten-year review of rating scales. II: scales for internalizing disorders J Am Acad Child Adolesc Psychiatry 2002 41 6 634 659 10.1097/00004583-200206000-00004 12049439

[B46] Reynolds WM Reynolds Adolescent depression Scale-2nd Edition: Short Form (RADS-2:SF) Professional Manual 2008 Lutz, FL: Psychological Assessment Resources, Inc

[B47] Beck S Beck Hopelessness Scale 1988 San Antonio: Psych Corporation

[B48] Beck AT Brown G Berchick RJ Stewart BL Steer RA Relationship between hopelessness and ultimate suicide: a replication with psychiatric outpatients Focus 2006 4 2 291 296 10.1176/ajp.147.2.190 2278535

[B49] March JS Parker JDA Sullivan K Stallings P Conners CK The Multidimensional Anxiety Scale for Children (MASC): factor structure, reliability, and validity J Am Acad Child Adolesc Psychiatry 1997 36 4 554 565 10.1097/00004583-199704000-00019 9100431

[B50] Robichaud M Dugas MJ Negative problem orientation (Part I): psychometric properties of a new measure Behav Res Ther 2005 43 3 391 401 10.1016/j.brat.2004.02.007 15680933

[B51] Jacob KL Christopher MS Neuhaus EC Development and validation of the Cognitive-Behavioral Therapy Skills Questionnaire Behav Modif 2011 35 6 595 618 10.1177/0145445511419254 21893554

[B52] Kraemer R Dzewaltowski D Blair M Rinehardt K Castracane V Mood alteration from treadmill running and its relationship to beta-endorphin, corticotropin and growth-hormone J Sport Med Phys Fit 1990 30 241 246 2176259

[B53] Berger B Grove J Prapavessis H Butki B Relationship of swimming distance, expectancy, and performance to mood states of competitive athletes Percept Motor Skill 1997 84 1199 1210 10.2466/pms.1997.84.3c.1199 9229436

[B54] Gould MS Marrocco FA Kleinman M Thomas JG Mostkoff K Cote J Davies M Evaluating iatrogenic risk of youth suicide screening programs: a randomized controlled trial JAMA 2005 293 13 1635 1643 10.1001/jama.293.13.1635 15811983

[B55] Yung AR Yuen HP McGorry PD Phillips LJ Kelly D Dell'Olio M Francey SM Cosgrave EM Killackey E Stanford C Godfrey K Buckby J Mapping the onset of psychosis: the Comprehensive Assessment of At-Risk Mental States Aust N Z J Psychiatry 2005 39 11–12 964 971 16343296 10.1080/j.1440-1614.2005.01714.x

[B56] Christie G Marsh R Sheridan J Wheeler A Suaalii-Sauni T Black S Butler R The Substances and Choices Scale Manual 2006 10.1111/j.1360-0443.2007.01916.x 17645425

[B57] Rickwood DJ Deane FP Wilson CJ When and how do young people seek professional help for mental health problems? Med J Aust 2007 187 7 S35 S39 17908023 10.5694/j.1326-5377.2007.tb01334.x

[B58] Robinson J Gook S Pan Yuen H McGorry P Yung Y Managing deliberate self-harm in young people: an evaluation of a training program developed for school welfare staff using a longitudinal research design BMC Psychiatry 2008 8 75 10.1186/1471-244X-8-75 PMC2564929 18789166

[B59] Slee N Garnefski N van der Leeden R Arensman E Spinhoven P Cognitive-behavioural intervention for self-harm: randomised controlled trial Br J Psychiatry 2008 192 3 202 211 10.1192/bjp.bp.107.037564 18310581

[B60] Robinson J Yuen H Gook S Hughes A Cosgrave E Killackey E Baker K Jorm A McGorry P Yung A Can receipt of a regular postcard reduce suicide-related behaviour in young help-seekers? A randomised controlled trial Early Interv Psychiatry 2012 6 2 145 152 10.1111/j.1751-7893.2011.00334.x 22260366

[B61] Goldney R Suicide Prevention: A Pragmatic Review of Recent Studies Crisis 2005 26 128 140 16276756 10.1027/0227-5910.26.3.128

[B62] March S Spence SH Donovan CL The efficacy of an internet-based cognitive-behavioral therapy intervention for child anxiety disorders J Pediatr Psychol 2009 5 474 487 18794187 10.1093/jpepsy/jsn099

[B63] Mathias C Furr M Sheftall A Hill-Kapturczak N Crum P Dougherty D What’s the harm in asking about suicidal ideation? Suicide Life Threat Behav 2012 42 3 341 351 10.1111/j.1943-278X.2012.0095.x 22548324 PMC3597074

[B64] Heckathorn D Respondent-driven sampling ii: deriving valid population estimates from chain-referral samples of hidden populations Soc Probl 2002 49 1 11 34 10.1525/sp.2002.49.1.11

